# Intraocular Pressure Increase Induced by 0.5% Tropicamide‐0.5% Phenylephrine in Dogs: An Analysis of Causes Using Ultrasound Biomicroscopy

**DOI:** 10.1111/vop.70015

**Published:** 2025-03-16

**Authors:** Donghee Kim, Sang‐Eun Park, Haerin Yoon, Jiyi Hwang, Ji Seung Jung, Soohyun Kim, Kyung‐Mee Park

**Affiliations:** ^1^ Laboratory of Veterinary Surgery and Ophthalmology College of Veterinary Medicine, Chungbuk National University Cheongju Korea; ^2^ Comparative Ophthalmology, Department of Surgical and Radiological Sciences, School of Veterinary Medicine University of California Davis Davis California USA

**Keywords:** anterior chamber anatomy, glaucoma risk, intraocular pressure increase, mydriatic agents, ultrasound biomicroscopy

## Abstract

**Purpose:**

To determine whether the differences in intraocular pressure (IOP) change after the topical application of 0.5% tropicamide and 0.5% phenylephrine (TP) are due to anatomical variations in the anterior chamber using ultrasound biomicroscopy (UBM).

**Methods:**

Prospective clinical data from 27 eyes of 14 dogs with normal eye conditions were analyzed. IOP and UBM measurements were taken before and 30 min after administering a topical TP. Dogs were categorized into two groups based on an IOP increase criterion of 4 mmHg: the high group and the stable group. Parameters measured included the iridocorneal angle (ICA), angle‐opening distance (AOD), ciliary cleft width (CCW), length (CCL), area (CCA), ciliary body axial length (CBAXL), and ciliary process‐sclera angle (CPSA).

**Results:**

Both groups showed a statistically significant decrease in ICA and an increase in AOD. However, in the stable group, CPSA increased, CBAXL decreased, and CCA increased, whereas in the high group, CPSA and CBAXL did not change significantly, and CCA decreased.

**Conclusion:**

In the stable group, although the pupil dilated, the ciliary body relaxed, leading to an increase in the CCA. Conversely, in the high group, the pupil dilated, and the ciliary body remained unrelaxed, resulting in a reduction in the CCA. These iridociliary changes with pupil dilation increase the risk of elevated IOP. Therefore, an increase in IOP following TP administration may serve as a prognostic indicator for possible glaucoma risk.

## 1 Introduction

The combination of 0.5% tropicamide and 0.5% phenylephrine is commonly utilized in veterinary ophthalmology to achieve effective pupil dilation (mydriasis), facilitating detailed ocular examinations and surgical interventions [1]. Tropicamide, a parasympatholytic agent, blocks muscarinic receptors in the iris sphincter muscle, leading to pupil dilation [2]. It also affects the ciliary body by inducing muscle relaxation [2]. Phenylephrine, a sympathomimetic agent, stimulates alpha‐1 adrenergic receptors in the iris dilator muscle, further enhancing mydriasis [3]. While phenylephrine has minimal direct effect on the ciliary body [4], the combined use of these agents results in a rapid and sustained pupil dilation, optimizing visualization of intraocular structures and supporting various diagnostic and therapeutic applications in veterinary ophthalmic practice [5].

One of the notable side effects of tropicamide is an increase in intraocular pressure (IOP) [6, 7, 8]. In one study, 68.8% of normal dogs experienced a rise in IOP, with the topical application of 0.5% tropicamide increasing IOP by approximately 8.8 ± 4.0 mmHg at 35 min post‐treatment compared to pre‐treatment levels [7]. Similarly, in human medicine, another study found that 68.9% of subjects showed an increase in IOP, with an average rise of 1.85 ± 2.01 mmHg [9]. Although the elevation in IOP induced by tropicamide is common, it does not occur in all individuals. A study in human medicine suggested that individual variations in IOP following pupil dilation could serve as a prognostic indicator for glaucoma. The research indicated that for every 1 mmHg increase in IOP after pupil dilation, the risk of glaucoma progression increases by 24% [10].

To investigate the causes of IOP increase following the use of mydriatic agents, the anatomical structures of the anterior segments have been studied using ultrasound biomicroscopy (UBM). Dulaurent et al. found that in dogs, the application of 0.5% tropicamide led to a reduction in the ciliary cleft (CC) width, increasing the risk of glaucoma [11]. Similarly, studies in humans have attributed the rise in IOP to a narrow iridocorneal angle (ICA) and the cycloplegic effect [6, 12, 13]. These studies commonly compare the anatomical structures before and after pupil dilation. Despite the variability in IOP increase among individuals after mydriatic application, to the best of the authors' knowledge, no research has analyzed the anatomical structures based on whether IOP increases or not.

The purpose of this study is to investigate the increase in IOP following the administration of a short‐acting combination of 0.5% tropicamide and 0.5% phenylephrine, which is commonly used in routine canine ophthalmic examinations due to its rapid onset and relatively brief duration of action compared to atropine or cyclopentolate. Additionally, this study aims to identify the anatomical variations in the anterior chamber that may contribute to this IOP elevation by using UBM. To the best of the authors' knowledge, this is the first study to examine the IOP increase following the application of this specific short‐acting mydriatic combination, as well as the first to explore the underlying anatomical causes of this increase through UBM analysis.

## 2 Materials and Methods

### 2.1 Clinical Information

This prospective study utilized clinical data from dogs admitted to the Veterinary Medical Teaching Hospital at Chungbuk National University in Cheongju, South Korea, between February 2023 and October 2023. The dataset comprised 27 eyes from 14 dogs. The measurements were taken from clinically normal dogs participating in a clinical trial, following consent from their guardians for academic and research purposes. Ethical approval for the study was granted by the Institutional Animal Care and Use Committee (CBNUA‐1700‐22‐02).

All dogs enrolled underwent thorough ophthalmological examinations conducted by a veterinary faculty member (KMP) or veterinarians specializing in ophthalmology (SEP). These included slit‐lamp biomicroscopy (MW50D, SHIGIYA, Hiroshima, Japan), the Schirmer Tear Test (Schirmer Tear Flow Strips, Gulden Ophthalmics, PA), and assessments of the menace response, pupillary light reflex, and dazzle reflex. Rebound tonometry was performed using the TonoVet Plus (icare, Vantaa, Finland). Additional procedures included gonioscopy (Ocular Koeppe Diagnostic Lenses, Ocular Instruments Inc., Bellevue, WA) and UBM using the VuPAD (Sonomed Escalon, Lake Success, NY), ensuring a thorough examination of canine ocular health.

### 2.2 Intra‐Ocular Pressure Measurement and UBM Examination

In our routine clinical practice, we regularly use a fixed combination of 0.5% tropicamide and 0.5% phenylephrine (Mydrin‐P; Alcon) to achieve effective pupil dilation for both diagnostic and therapeutic purposes. Accordingly, in this study, IOP measurements and UBM examinations were conducted immediately before and 30 min after administering a single drop of this combination agent.

The IOP was measured at each time point using rebound tonometry (Tonovet Plus, icare) with the dogs in a seated position. Thirty minutes after eye drop instillation, pupil dilation was confirmed in all participants prior to IOP measurement. Topical anesthesia was administered using 0.5% proparacaine hydrochloride (Alcaine; Alcon). During the UBM procedure, care was taken to avoid applying pressure to the globe while ensuring clear visualization of the dorsal quadrant of the eye. The UBM probe was positioned perpendicularly to the corneoscleral limbus in this quadrant to obtain accurate imaging.

**FIGURE 1:**
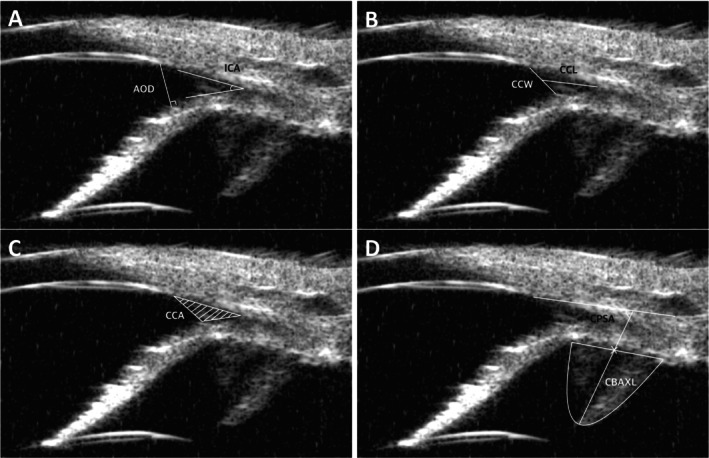
UBM examination parameters. (A) Measurement of the ICA and AOD. The ICA is identified at the peripheral circumferential section where the sclera, cornea, and iris base meet, with the angle measured between the inner layer of the sclera and the outer layer of the iris root. The AOD is assessed by drawing a perpendicular line from the endpoint of Descemet's membrane to the anterior surface of the iris. (B) CCW is measured as the distance from where the outer layer of the pectinate ligament meets the inner boundary of the sclera to the upper surface of the iris root. CCL is determined by measuring the distance from the angle recess to the midpoint of the CCW. (C) CCA is calculated by mapping the area formed by tracing the inner scleral surface from the angle recess to its inner boundary and the superior surface of the iris root up to the CCW. (D) CBAXL is determined by measuring the distance from the apex of the dome‐shaped ciliary body to the center of the ciliary body along its long axis. The CPSA is defined as the angle between the long axis of the ciliary body and the sclera.

In this study, a total of eight parameters were used. First, the ICA and the angle‐opening distance (AOD) were measured to represent the angle and size of the entrance through which aqueous humor flows. The geometric ICA was identified by the peripheral circumferential section where the sclera, cornea, and iris base meet. This section served as a reference point for drawing lines along the inner layer of the sclera and the outer layer of the iris root, with the angle between these two lines being measured [11, 14]. The AOD was assessed by drawing a perpendicular line from the endpoint of Descemet's membrane to the anterior surface of the iris, providing a quantifiable measure of the ICA's openness (Figure 1A) [15, 16].

Secondly, three CC parameters were evaluated by assessing the CC width (CCW), CC length (CCL), and CC area (CCA). The CCW was measured as the distance from the point where the outer layer of the pectinate ligament meets the inner boundary of the sclera to the point where it reaches the upper surface of the iris root. The CCL was determined by measuring the distance from the angle recess to the midpoint of the CCW (Figure 1B). The CCA was calculated by mapping the area formed by tracing the inner scleral surface from the angle recess to its inner boundary and the superior surface of the iris root up to the CCW, providing detailed insights into the anatomical structure of the CC (Figure 1C) [14, 17, 18].

Finally, the study included measurements of two ciliary body parameters: the axial length of the ciliary body (CBAXL) and the ciliary process‐sclera angle (CPSA). The CBAXL was determined by drawing a line through the apex of the dome‐shaped ciliary body to the center of the ciliary body, measured manually using the UBM's built‐in software. This length was calculated as the distance between the apex of the ciliary body and the uveoscleral interface along its long axis. The CPSA was defined as the angle between the long axis of the ciliary body and the sclera (Figure 1D) [19, 20]. These detailed measurements, taken using the UBM's integrated software and averaged from at least three separate images, ensured precise and reliable data on the ciliary body's involvement in ocular health.

To minimize bias, the UBM examinations were conducted by SEP, while the analysis of the UBM measurements, using the device's built‐in software, was performed by a different individual who was blinded to the patient groups. This individual was unaware of the patients' identities during the analysis, ensuring the objectivity of the measurements.

### 2.3 Patient Groups

In this study, we categorized the patients into different groups based on the timing of topical tropicamide‐phenylephrine (TP) administration and IOP changes observed. The pre‐TP group consisted of patients evaluated before the administration of topical 0.5% tropicamide and 0.5% phenylephrine, with baseline measurements including UBM and IOP. The post‐TP group included patients who were evaluated after the administration of topical 0.5% tropicamide and 0.5% phenylephrine, using the same methods as in the pre‐TP group to assess changes in the anterior chamber and IOP. Referring to previous research findings, patients whose IOP increased by 4 mmHg or more after the administration of topical TP were categorized into the high group [9, 21]. The stable group consisted of patients whose IOP did not increase by 4 mmHg or more following the administration of topical TP, serving as a control to compare with those experiencing significant IOP changes.

### 2.4 Statistical Analysis

In this investigation, statistical analyses were executed utilizing SPSS software (version 17.0; SPSS Inc., Chicago) to delve into the nuances of our data. Initially, several statistical methods were employed to analyze factors affecting the comparison between the high group and the stable group. The Chi‐squared (*χ* ^2^) test was applied to examine differences across breed and categories among the subjects. Additionally, to verify differences in age and weight, the Shapiro–Wilk test was conducted to determine the normality of the data set. Following confirmation of data normality, the *t*‐test was leveraged to compare mean values. Furthermore, the t‐test was used to compare baseline IOP between the high and stable groups, as well as to compare IOP and various parameters before and after the administration of TP within the high and stable groups. For visual clarity in the presentation of results, asterisks are utilized in the figures, while precise *p*‐values are thoroughly detailed within the study's text.

## 3 Results

### 3.1 Canine Demography

The stable group consisted of 10 dogs and 17 eyes. Shih Tzus (*n* = 3) were the most common breed, followed by Pomeranians (*n* = 2), with additional breeds including Poodles, Spitz, Maltese, Beagles, and mixed breeds. The average age of the dogs in the stable group was 8.14 ± 4.96 years, with a range from 2 to 15 years. The average weight of the stable group was 5.32 ± 1.88 kg, ranging from 2.85 to 8.30 kg.

The high group consisted of 7 dogs and 10 eyes. All breeds were represented by a single dog each, including Cavalier king charles spaniel, Spitz, Beagle, Chihuahua, Maltese, Pomeranian, and Shih Tzu. The dogs in the high group had an average age of 9.43 ± 3.01 years, spanning from 6 to 15 years. The average weight of the dogs in the high group was 5.66 ± 1.21 kg, with a range from 4.40 to 8.00 kg.

Statistical analyses were conducted to compare age and weight between the stable and high groups. These analyses revealed no significant differences in these parameters, indicating a comparable distribution of age (*p* = 0.42) and weight (*p* = 0.58) across both groups.

### 3.2 IOP Changes After Topical Application in the Entire Experimental Group

The IOP measurements before the application of topical TP had a mean of 17.00 ± 3.16 mmHg, ranging from 10 to 22 mmHg. After the application, the IOP had a mean of 19.15 ± 3.20 mmHg, ranging from 14 to 27 mmHg. The mean difference in IOP before and after the application was 2.15 ± 3.46 mmHg (Figure 3A). These results indicate a statistically significant increase in IOP following the application (*p* = 0.0021).

**FIGURE 2:**
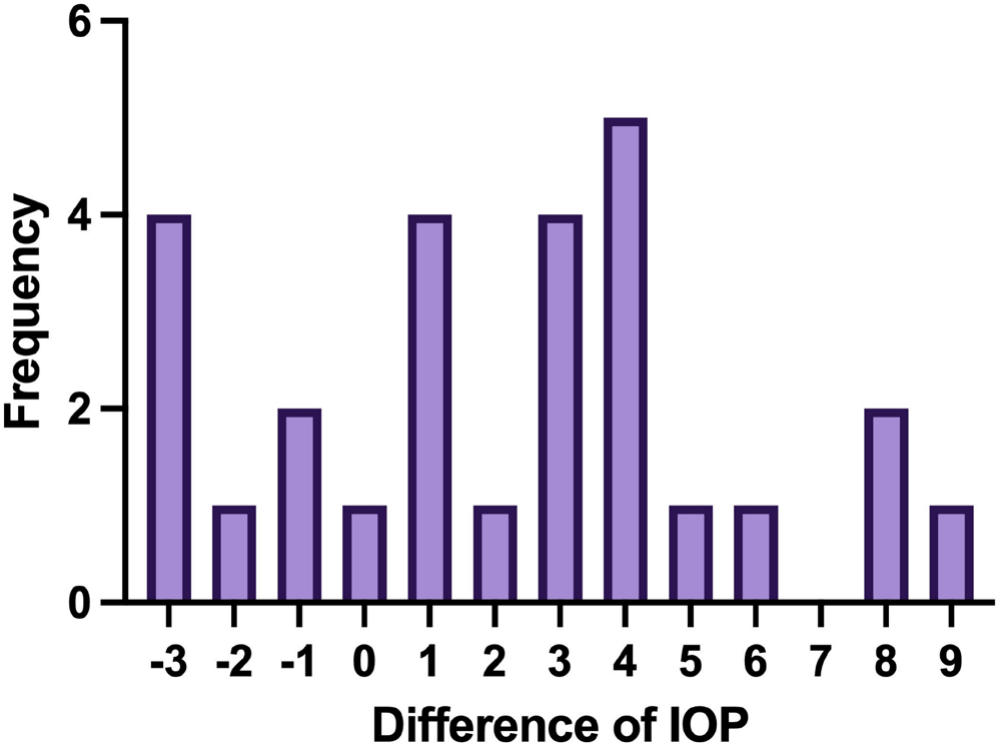
Frequency distribution of IOP differences after topical application. The distribution of IOP differences before and after the application of topical 0.5% tropicamide and 0.5% phenylephrine in the entire experimental group (*n* = 27). Differences below 4 mmHg were more common, with differences of −3 and 1 mmHg observed in 4 cases each, and differences of 2 and 3 mmHg observed in 4 cases each. Differences of 4 and above were less frequent, with a 4 mmHg difference observed in 4 cases and differences of 5, 7, 8, and 9 mmHg in fewer cases.

The frequency distribution of IOP differences indicated that variations below 4 mmHg were more common. Specifically, differences of −3, 1, and 3 mmHg were each observed in 4 cases, while differences of −1 and 8 mmHg were seen in 2 cases each. Less frequently, differences of −2, 0, 2, 5, 6, and 9 mmHg were each observed in 1 case. These results suggest that IOP differences around 3–4 mmHg were the most prevalent in this study (Figure 2).

### 3.3 IOP In the Stable and High Groups

To determine the difference in baseline IOP between the groups, we compared the pre‐TP values. The stable group (*n* = 17) had a mean IOP of 17.76 ± 2.51 mmHg, ranging from 14 to 22 mmHg. The high group (*n* = 10) had a mean IOP of 15.70 ± 3.83 mmHg, with a range of 10 to 22 mmHg. Statistical analysis revealed no significant difference in baseline IOP between the stable and high groups (*p* = 0.1022).

**FIGURE 3:**
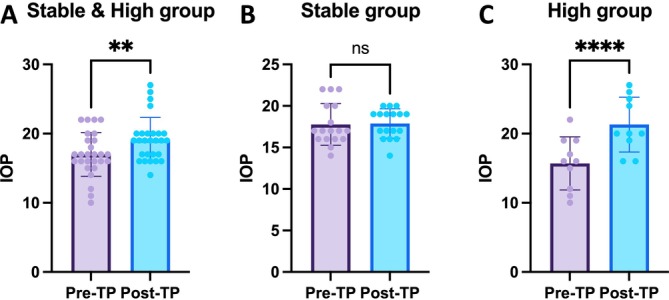
IOP changes in stable and high groups. (A) Mean IOP in the Stable & High group (*n* = 27) before and after the application of topical 0.5% tropicamide and 0.5% phenylephrine. Pre‐TP IOP: 17.00 ± 3.16 mmHg; Post‐TP IOP: 19.15 ± 3.20 mmHg. The mean difference in IOP was 2.15 ± 3.46 mmHg, showing a significant increase (p < 0.01). (B) Mean IOP in the stable group (*n* = 17) before and after the application of topical 0.5% tropicamide and 0.5% phenylephrine. Pre‐TP IOP: 17.76 ± 2.51 mmHg; Post‐TP IOP: 17.88 ± 1.76 mmHg. The mean difference in IOP was 0.12 ± 2.32 mmHg, with no significant change (*p* = 0.8367). (C) Mean IOP in the high group (*n* = 10) before and after the application. Pre‐TP IOP: 15.70 ± 3.83 mmHg; Post‐TP IOP: 21.30 ± 3.97 mmHg. The mean difference in IOP was 5.60 ± 2.01 mmHg, showing a significant increase (*p* < 0.0001). **Indicates *p* < 0.05, **represents *p* < 0.01, ***denotes *p* < 0.001, and ****signifies *p* < 0.0001.

In the stable group, the pre‐TP IOP had a mean of 17.76 ± 2.51 mmHg, ranging from 14 to 22 mmHg, while the post‐TP IOP had a mean of 17.88 ± 1.76 mmHg, ranging from 14 to 20 mmHg. The mean difference in IOP was 0.12 ± 2.32 mmHg. Statistical analysis revealed no significant difference in IOP between the Pre‐TP and Post‐TP measurements (*p* = 0.8367) (Figure 3B).

In the high group, the pre‐TP IOP had a mean of 15.70 ± 3.83 mmHg, ranging from 10 to 22 mmHg, while the post‐TP IOP had a mean of 21.30 ± 3.97 mmHg, ranging from 16 to 27 mmHg. The mean difference in IOP was 5.60 ± 2.01 mmHg. Statistical analysis revealed a significant increase in IOP between the pre‐TP and post‐TP measurements (*p* < 0.0001) (Figure 3C).

### 3.4 Anterior Chamber Parameters Change After Topical Application in the Stable Group

In the stable group, the anterior parameters of the anterior chamber were analyzed before and after the application of topical TP.

#### 3.4.1 ICA and AOD Changes

**FIGURE 4:**
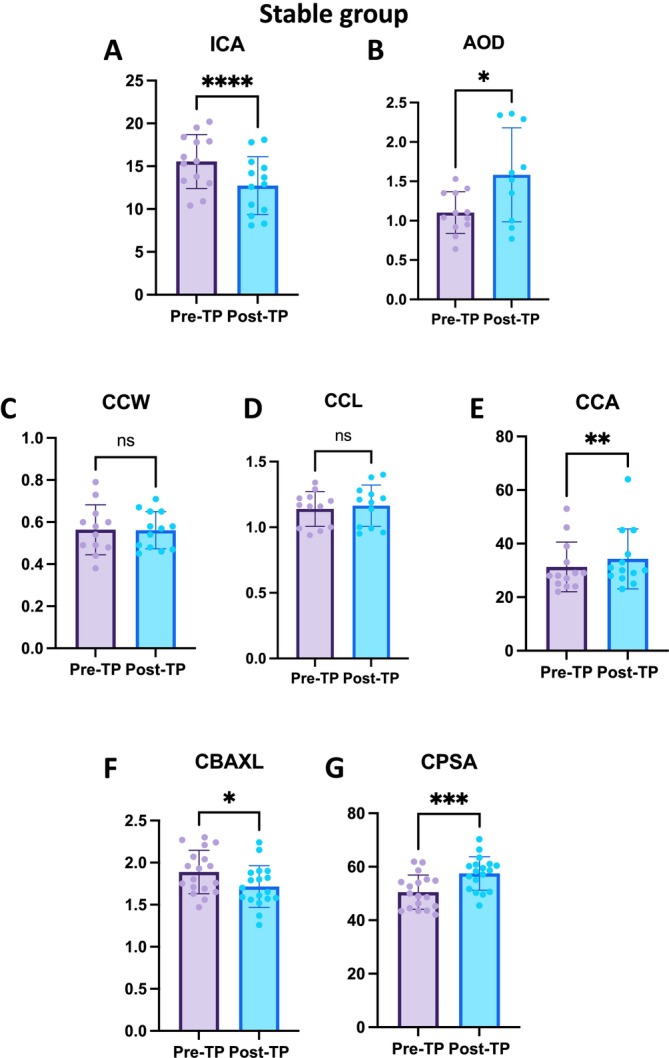
Anterior chamber parameter changes after topical application in the stable group. (A) ICA changes in the stable group (*n* = 17). Pre‐TP ICA: 15.55° ± 3.15°; Post‐TP ICA: 12.74° ± 3.37°. Significant decrease in ICA (*p* < 0.0001). (B) AOD changes in the stable group. Pre‐TP AOD: 1.10 ± 0.26 mm; Post‐TP AOD: 1.58 ± 0.60 mm. Significant increase in AOD (*p* = 0.0137). (C) CCW changes in the stable group. Pre‐TP CCW: 0.56 ± 0.12 mm; Post‐TP CCW: 0.56 ± 0.09 mm. No significant change (*p* = 0.9151). (D) CCL changes in the stable group. Pre‐TP CCL: 1.139 ± 0.13 mm; Post‐TP CCL: 1.164 ± 0.16 mm. No significant change (*p* = 0.2691). (E) CCA changes in the stable group. Pre‐TP CCA: 31.31 ± 9.28 mm^2^; Post‐TP CCA: 34.31 ± 11.18 mm^2^. Significant increase in CCA (*p* = 0.0036). (F) CBAXL changes in the stable group. Pre‐TP CBAXL: 1.889 ± 0.258 mm; Post‐TP CBAXL: 1.716 ± 0.248 mm. Significant decrease in CBAXL (*p* = 0.0417). (G) CPSA changes in the stable group. Pre‐TP CPSA: 50.52° ± 6.397°; Post‐TP CPSA: 57.51° ± 6.274°. Significant increase in CPSA (*p* = 0.0004). *Indicates *p* < 0.05, **represents *p* < 0.01, ***denotes *p* < 0.001, and ****signifies *p* < 0.0001.

For the ICA, the pre‐TP ICA had a mean of 15.55° ± 3.15°, ranging from 10.40°to 20.20°. The post‐TP ICA had a mean of 12.74° ± 3.37°, with a range from 8.10° to 18.10°. The mean difference in ICA was −2.82° ± 1.51°. Statistical analysis revealed a significant decrease in ICA following the application (*p* < 0.0001) (Figure 4A).

For the AOD, the pre‐TP AOD had a mean of 1.10 ± 0.26 mm, ranging from 0.64 to 1.53 mm. The post‐TP AOD had a mean of 1.58 ± 0.60 mm, with a range from 0.77 to 2.36 mm. The mean difference in AOD was 0.52 ± 0.54 mm. Statistical analysis revealed a significant increase in AOD following the application (*p* < 0.05) (Figure 4B).

The decrease in ICA and the increase in AOD indicate that the iris has undergone dilation.

#### 3.4.2 CC Changes

For the CCW, the pre‐TP CCW had a mean of 0.56 ± 0.12 mm, ranging from 0.38 to 0.79 mm. The post‐TP CCW had a mean of 0.56 ± 0.09 mm, with a range from 0.45 to 0.71 mm. The mean difference in CCW was −0.003 ± 0.11 mm. Statistical analysis revealed no significant difference in CCW following the application (*p* = 0.9151) (Figure 4C).

For the CCL, the pre‐TP CCL had a mean of 1.14 ± 0.13 mm, ranging from 0.94 to 1.34 mm. The post‐TP CCL had a mean of 1.16 ± 0.16 mm, with a range from 0.95 to 1.40 mm. The mean difference in CCL was 0.025 ± 0.07 mm. Statistical analysis revealed no significant difference in CCL following the application (*p* = 0.2691) (Figure 4D).

For the CCA, the pre‐TP CCA had a mean of 31.31 ± 9.28 mm^2^, ranging from 22.00 to 53.00 mm^2^. The post‐TP CCA had a mean of 34.31 ± 11.18 mm^2^, with a range from 23.00 to 64.00 mm^2^. The mean difference in CCA was 3.00 ± 3.00 mm^2^. Statistical analysis revealed a significant increase in CCA following the application (*p* < 0.01) (Figure 4E).

For CCW and CCL, there were no statistically significant changes observed. However, the values for CCL did show an increase. Overall, given that CCA increased significantly, it appears that the area controlling the flow of aqueous humor has expanded.

#### 3.4.3 Ciliary Body Changes

For the CBAXL, the pre‐TP CBAXL had a mean of 1.89 ± 0.26 mm, ranging from 1.47 to 2.30 mm. The post‐TP CBAXL had a mean of 1.72 ± 0.25 mm, with a range from 1.26 to 2.24 mm. The mean difference in CBAXL was −0.17 ± 0.33 mm. Statistical analysis revealed a significant decrease in CBAXL following the application (*p* < 0.05) (Figure 4F).

For the CPSA, the pre‐TP CPSA had a mean of 50.52° ± 6.397°, ranging from 42.10° to 61.90°. The post‐TP CPSA had a mean of 57.51° ± 6.27°, with a range from 45.50° to 70.30°. The mean difference in CPSA was 6.99° ± 6.74°. Statistical analysis revealed a significant increase in CPSA following the application (*p* < 0.001) (Figure 4G).

The decrease in CBAXL and the increase in CPSA indicate that the ciliary body moved inward and anteriorly, respectively, which is an indicator of ciliary body relaxation.

### 3.5 Anterior Chamber Parameters Change After Topical Application in the High Group

In the high group, the anterior chamber parameters of the anterior chamber were analyzed before and after the application of topical TP.

#### 3.5.1 ICA And AOD Changes

**FIGURE 5:**
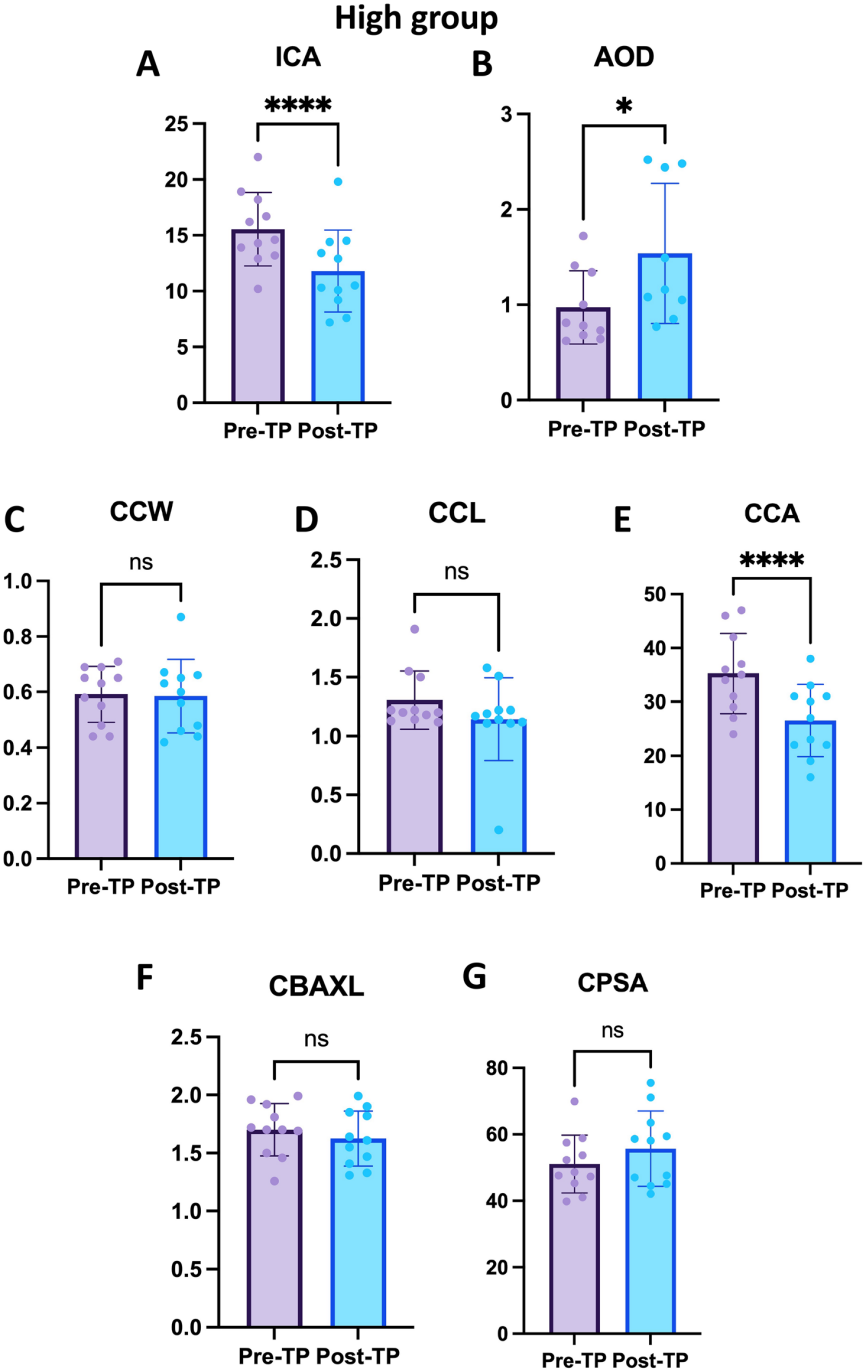
Anterior chamber parameter changes after topical application in the high group. (A) ICA changes in the high group (*n* = 10). Pre‐TP ICA: 15.79° ± 3.36°; Post‐TP ICA: 12.27° ± 3.51°. Significant decrease in ICA (*p* < 0.0001). (B) AOD changes in the high group. Pre‐TP AOD: 0.97 ± 0.38 mm; Post‐TP AOD: 1.54 ± 0.74 mm. Significant increase in AOD (*p* < 0.05). (C) CCW changes in the high group. Pre‐TP CCW: 0.603 ± 0.099 mm; Post‐TP CCW: 0.600 ± 0.130 mm. No significant change (*p* = 0.9293). (D) CCL changes in the high group. Pre‐TP CCL: 1.317 ± 0.257 mm; Post‐TP CCL: 1.135 ± 0.369 mm. No significant change (*p* = 0.3128). (E) CCA changes in the high group. Pre‐TP CCA: 36.10 ± 7.31 mm^2^; Post‐TP CCA: 27.30 ± 6.53 mm^2^. Significant decrease in CCA (*p* = 0.0003). (F) CBAXL changes in the high group. Pre‐TP CBAXL: 1.745 ± 0.180 mm; Post‐TP CBAXL: 1.655 ± 0.227 mm. No significant change (*p* = 0.3202). (G) CPSA changes in the high group. Pre‐TP CPSA: 49.19° ± 6.397°; Post‐TP CPSA: 54.90° ± 11.62°. No significant change (*p* = 0.1902). * Indicates *p* < 0.05, ** represents *p* < 0.01, *** denotes *p* < 0.001, and **** signifies *p* < 0.0001.

For the ICA, the pre‐TP ICA had a mean of 15.79° ± 3.36°, ranging from 10.20° to 22.00°. The post‐TP ICA had a mean of 12.27° ± 3.51°, with a range from 7.60° to 19.80°. The mean difference in ICA was −3.52° ± 1.55°. Statistical analysis revealed a significant decrease in ICA following the application (*p* < 0.0001) (Figure 5A).

For the AOD, the pre‐TP AOD had a mean of 0.97 ± 0.38 mm, ranging from 0.62 to 1.72 mm. The post‐TP AOD had a mean of 1.54 ± 0.74 mm, with a range from 0.77 to 2.52 mm. The mean difference in AOD was 0.56 ± 0.26 mm. Statistical analysis revealed a significant increase in AOD following the application (*p* < 0.05) (Figure 5B).

The decrease in ICA and the increase in AOD indicate that the iris has undergone dilation. This result was consistent with findings in the stable group.

#### 3.5.2 CC Changes

For the CCW, the pre‐TP CCW had a mean of 0.60 ± 0.10 mm, ranging from 0.44 to 0.71 mm. The post‐TP CCW had a mean of 0.60 ± 0.13 mm, with a range from 0.42 to 0.87 mm. The mean difference in CCW was −0.003 ± 0.10 mm. Statistical analysis revealed no significant difference in CCW following the application (*p* = 0.9293). (Figure 5C).

For the CCL, the pre‐TP CCL had a mean of 1.32 ± 0.26 mm, ranging from 1.12 to 1.91 mm. The post‐TP CCL had a mean of 1.14 ± 0.37 mm, with a range from 0.20 to 1.58 mm. The mean difference in CCL was −0.18 ± 0.54 mm. Statistical analysis revealed no significant difference in CCL following the application (*p* = 0.3128) (Figure 5D).

For the CCA, the pre‐TP CCA had a mean of 36.10 ± 7.31 mm^2^, ranging from 24.00 to 47.00 mm^2^. The post‐TP CCA had a mean of 27.30 ± 6.53 mm^2^, with a range from 16.00 to 38.00 mm^2^. The mean difference in CCA was −8.80 ± 4.87 mm^2^. Statistical analysis revealed a significant decrease in CCA following the application (*p* < 0.001) (Figure 5E).

While there were no statistically significant changes in CCW and CCL, CCA significantly decreased. This indicates that the anatomical area controlling the flow of aqueous humor has reduced in size.

#### 3.5.3 Ciliary Body Changes

For the CBAXL, the pre‐TP CBAXL had a mean of 1.75 ± 0.18 mm, ranging from 1.46 to 1.99 mm. The post‐TP CBAXL had a mean of 1.66 ± 0.23 mm, with a range from 1.31 to 1.99 mm. The mean difference in CBAXL was −0.09 ± 0.27 mm. Statistical analysis revealed no significant difference in CBAXL following the application (*p* = 0.3202) (Figure 5F).

For the CPSA, the pre‐TP CPSA had a mean of 49.19° ± 6.40°, ranging from 39.80° to 58.80°. The post‐TP CPSA had a mean of 54.90° ± 11.62°, with a range from 42.10° to 75.50°. The mean difference in CPSA was 5.71° ± 11.62°. Statistical analysis revealed no significant difference in CPSA following the application (*p* = 0.1902) (Figure 5G).

The results for CBAXL and CPSA indicate that there was neither contraction nor relaxation of the ciliary body.

## 4 Discussion

The purpose of this study was to observe structural changes in the anterior chamber and to assess the corresponding changes in IOP following pupil dilation. To achieve this, subjects were divided into two groups based on the degree of IOP increase before and after the topical application of a combination of 0.5% tropicamide and 0.5% phenylephrine. The key differentiating factors between the two groups were the CC and the ciliary body. In the high group, CCA decreased, and the ciliary body did not relax; in contrast, the stable group showed an increase in CCA and relaxation of the ciliary body. These findings suggest that the CC plays a primary role in IOP elevation, with changes in the ciliary body contributing to alterations in CC.

In the present study, IOP and UBM were measured 30 min after administering the TP drop. This decision was based on the findings of Kovalcuka et al. which reported that IOP peaked at approximately 30–35 min following 0.5% tropicamide instillation. In the same study, although IOP peaked at 10 min when using 10% phenylephrine, the increase was not statistically significant [7]. Moreover, a human study comparing 1% tropicamide alone with a 1% tropicamide–2.5% phenylephrine combination found no significant difference in IOP between the two regimens at 30–40 min post‐instillation [22]. In the present work, we employed 0.5% phenylephrine, which is substantially lower than either 10% or 2.5%, thereby likely exerting a negligible impact on IOP. Additionally, we confirmed that maximal mydriasis was also achieved at this time point. Consequently, we focused on the 30‐min time point—when 0.5% tropicamide typically induces its greatest IOP elevation and pupil dilation—to capture the maximal effect for both IOP and UBM measurements.

This study observed changes in the CC in response to the effects of TP and to explain the inconsistent IOP changes following the application of these topical medications. The CC plays a crucial role in IOP regulation, and its reduction is known to increase the risk of glaucoma [18, 23]. The CC is influenced by two adjacent anatomical structures: first, the dilation of the iris, which structurally compresses the CC, and second, the contraction of the ciliary body, which also compresses the CC [9, 11, 17, 20]. The medications used in this study, TP, are known to dilate the iris, while tropicamide also relaxes the ciliary body [7, 24, 25]. In other words, these medications simultaneously have two opposing effects on the CC: dilating the iris to enlarge the CC and relaxing the ciliary body to reduce the CC. We hypothesized that the balance between these opposing actions determines the size of the CC, and this difference accounts for the variations in IOP following medication application.

Although the increases in IOP observed in our study remained within the normal reference interval for dogs, we considered them clinically significant due to the potential implications of IOP fluctuations on ocular health and glaucoma risk assessment [10, 21]. We defined IOP variability as diurnal IOP variation and adopted a threshold of 4 mmHg, which corresponds to the normal diurnal IOP variation in dogs [26]. Additionally, human studies have reported that diurnal IOP variation in normal subjects ranges from approximately 2 to 6 mmHg, with an average of 4 mmHg [9]. This approach is methodologically consistent with previous human medical studies that considered IOP fluctuations exceeding the normal diurnal variation as significant [21]. By setting our cutoff at 4 mmHg, we aimed to identify IOP fluctuations that may indicate underlying variations in anterior chamber dynamics. Even within normal limits, such IOP fluctuations can be an independent risk factor for glaucoma progression [10]. Therefore, analyzing these fluctuations enhances our understanding of how IOP variability affects the eye and underscores the importance of considering not only absolute IOP values but also the magnitude of IOP fluctuations in evaluating ocular health.

In this study, there was a difference in the relaxation of the ciliary body between the two groups. Tropicamide, an anticholinergic agent, and phenylephrine, a sympathomimetic agent, collectively contribute to the relaxation of the ciliary body [12, 27]. In the stable group, the decrease in CBAXL and the increase in CPSA indicate that the ciliary body was relaxed [19, 20, 28]. Although the changes in CCW and CCL were not significant, the increase in CCA suggests that the effect of the ciliary body relaxation, which enlarges the CC, outweighed the effect of iris dilation, which reduces the CC. Conversely, in the high group, there were no significant changes in the ciliary body parameters. It is likely that only the effect of iris dilation, which reduces the CC, was present, leading to a decrease in CCA. The resulting changes in the CCA can explain the IOP variations observed in each group [18, 29].

Despite identical study conditions, the difference in the response of the ciliary body between the groups suggests a predisposition to glaucoma. Previous research has shown that in eyes with acute angle‐closure glaucoma and their fellow eyes, the ciliary bodies were thinner and more anteriorly rotated, indicating contraction of the ciliary body [20, 30]. In this study, even with the use of topical anticholinergic medication, the ciliary body did not relax in the high group. This could be due to the ciliary body being contracted and unable to relax due to a predisposition to glaucoma, likely accounting for the observed variations in IOP increase among individuals. Additionally, the contraction of the ciliary body may reduce the unconventional outflow pathway, further contributing to the increase in IOP [31, 32, 33]. Thus, the inability of the ciliary body to relax, due to its contracted state and the predisposition to glaucoma, may explain the differences in IOP response to the medication among the subjects.

Change in IOP with pupillary dilation is a form of IOP fluctuation. It is reasonable to assume that eyes exhibiting changes in IOP following dilation experience larger‐thanthan‐usual fluctuations in IOP at other times. Previous research has demonstrated that the extent of IOP increase due to pharmacological pupillary dilation is related to the likelihood of future glaucoma progression [10, 34, 35]. This study found that such IOP fluctuations are caused by the inability of the ciliary body to relax, a condition similar to that observed in the eyes of individuals with glaucoma and their fellow eyes [30]. Given these findings, it is appropriate to consider an increase in IOP following dilation as a potential prognostic indicator for the development of glaucoma.

Contrary to previous beliefs in veterinary medicine, it appears that the ICA also plays a significant role in IOP formation [11, 17]. In the stable group, although the CC significantly expanded, IOP did not decrease; in fact, there was no significant change in IOP. This suggests that the reduction in ICA may have counteracted the effects of the expanded CC, leading to resistance in the aqueous humor outflow and stabilizing the IOP.

These findings suggest that the ICA plays a critical role in IOP elevation, aligning with human medical studies that indicate the narrowing of the ICA following dilation can cause crowding in the angle, subsequently disrupting the trabecular meshwork's outflow facility and leading to an increase in IOP [6]. However, previous veterinary studies suggest that the geometric angle might be less significant in dogs compared to humans regarding glaucoma risk, as the trabecular meshwork in dogs is exposed differently, making it a less likely risk factor for glaucoma [11, 17]. Nonetheless, based on the results of this study, it appears that the ICA also plays an important role in IOP regulation in dogs.

In this study, the degree of IOP increase following the use of mydriatic agents differed from previous studies. In our research, the IOP increased by an average of 2.15 ± 3.46 mmHg across all patients. In contrast, Kovalcuka et al. reported an 8.8 ± 4.0 mmHg increase in IOP in normal dogs after using 0.5% tropicamide alone, and Katrin et al. observed a 3.8 ± 4.2 mmHg increase in normal cats [7, 8]. The smaller IOP increase in our study might be attributed to the difference in the medications used. We used a combination of 0.5% tropicamide and 0.5% phenylephrine, whereas the other studies used 0.5% tropicamide alone. Although low‐dose phenylephrine is not known to relax the ciliary body, the combination of the two drugs might have produced a synergistic effect, leading to greater relaxation of the ciliary body and, consequently, a smaller increase in IOP compared to the use of 0.5% tropicamide alone [24, 36]. Based on these observations, it may be beneficial to relax the ciliary body when dilation is needed for ophthalmic examinations, as this could reduce the extent of IOP increase and ensure a safer examination. Using a combination eye drop that includes 0.5% phenylephrine or increasing the dosage to elevate the drug concentration in the anterior chamber may result in more effective relaxation of the ciliary body and a reduced increase in IOP [37].

Previous human studies have suggested that the mechanism for increased IOP following the use of mydriatic agents may be related to decreased aqueous outflow due to reduced traction on the trabecular meshwork caused by ciliary muscle paralysis [12, 13, 27]. However, due to anatomical differences in the position of the trabecular meshwork, it is hypothesized that in dogs, ciliary muscle paralysis—or relaxation of the ciliary muscle—actually widens the ciliary cleft (the canine equivalent of the trabecular meshwork complex), thereby lowering IOP. Additionally, in humans, the application of tropicamide has been associated with increased pigment accumulation in the anterior chamber, which is thought to partially obstruct the drainage structures [38, 39]. However, this phenomenon was not observed in our study.

In this study, tropicamide and phenylephrine were used, which are known to have a limited ability to induce cycloplegia and, therefore, are less effective at relaxing the ciliary body [40]. This may have contributed to the variability in ciliary body relaxation among individuals, leading to a wide distribution of IOP differences between pre‐TP and post‐TP measurements. Based on these findings, it is hypothesized that using atropine, a pupil dilator with a stronger cycloplegic effect, would result in a narrower distribution of IOP differences and more consistent outcomes [41]. Conducting a study using atropine could provide a deeper understanding of the relationship between pupil dilation, IOP elevation, and the development of glaucoma.

Several limitations should be considered in this study. Firstly, the sample size was relatively small, encompassing only 27 eyes from 14 dogs. This limited sample size may affect the generalizability of the results and reduce the statistical power to detect subtle differences between the stable and high groups. Furthermore, the study did not follow up with the high group to determine if the increased IOP indeed progressed to glaucoma over time, which limits the ability to confirm the predictive value of IOP increase as an indicator of glaucoma development. Longitudinal studies would be beneficial to confirm these findings, assess the long‐term implications of IOP fluctuations on glaucoma progression, and verify whether the observed anatomical changes lead to clinical glaucoma.

In this study, we were unable to establish a correlation between gonioscopy findings and IOP elevation, which represents a limitation. We included only individuals with open ICA as determined by gonioscopy, excluding those with narrow angles and pectinate ligament dysplasia (PLD). As a result, we did not observe the effects of narrow angles or PLD on IOP changes within our study population. Including subjects with narrow angles or PLD could have provided additional insights into their potential contribution to IOP elevation. Future studies that incorporate a broader range of gonioscopic findings may yield a more comprehensive understanding of the anatomical factors influencing IOP changes.

In our study, we selected the combination of 0.5% tropicamide and 0.5% phenylephrine, commercially known as Mydrin‐P, which is a mydriatic mix commonly used in veterinary ophthalmology in Asia for effective pupil dilation. This choice was guided by the combination's established efficacy, rapid onset, and shorter duration of action in canine clinical settings, making it suitable for routine ophthalmic examinations without causing prolonged mydriasis that could be inconvenient for patients and owners [7]. While alternatives like atropine and cyclopentolate are potent mydriatics, their long‐lasting dilation effects can be disadvantageous in general practice, leading us to opt for Mydrin‐P [7, 42]. Each mydriatic agent interacts differently with ocular structures, potentially affecting the ciliary body and CC, and consequently influencing IOP.

A limitation of this study is that we did not include separate groups receiving only 0.5% tropicamide or only 0.5% phenylephrine, which prevents us from fully assessing the potential boosting effect of 0.5% phenylephrine on tropicamide‐induced mydriasis. However, we chose to use this combined formulation because, in our country and certain other regions, it is routinely prescribed, whereas using two separate agents in different concentrations is relatively rare. Consequently, our primary aim was to provide clinically relevant data for conditions commonly encountered in actual practice. Nonetheless, future studies that incorporate each agent independently and measure IOP at multiple time points would offer a more comprehensive view of the dynamic interactions among IOP, the ciliary cleft, and ciliary body changes. Such an approach could yield deeper insights into the extent to which low‐dose phenylephrine contributes to IOP regulation and pupil dilation, ultimately improving clinical protocols for canine ophthalmic examinations.

**FIGURE 6:**
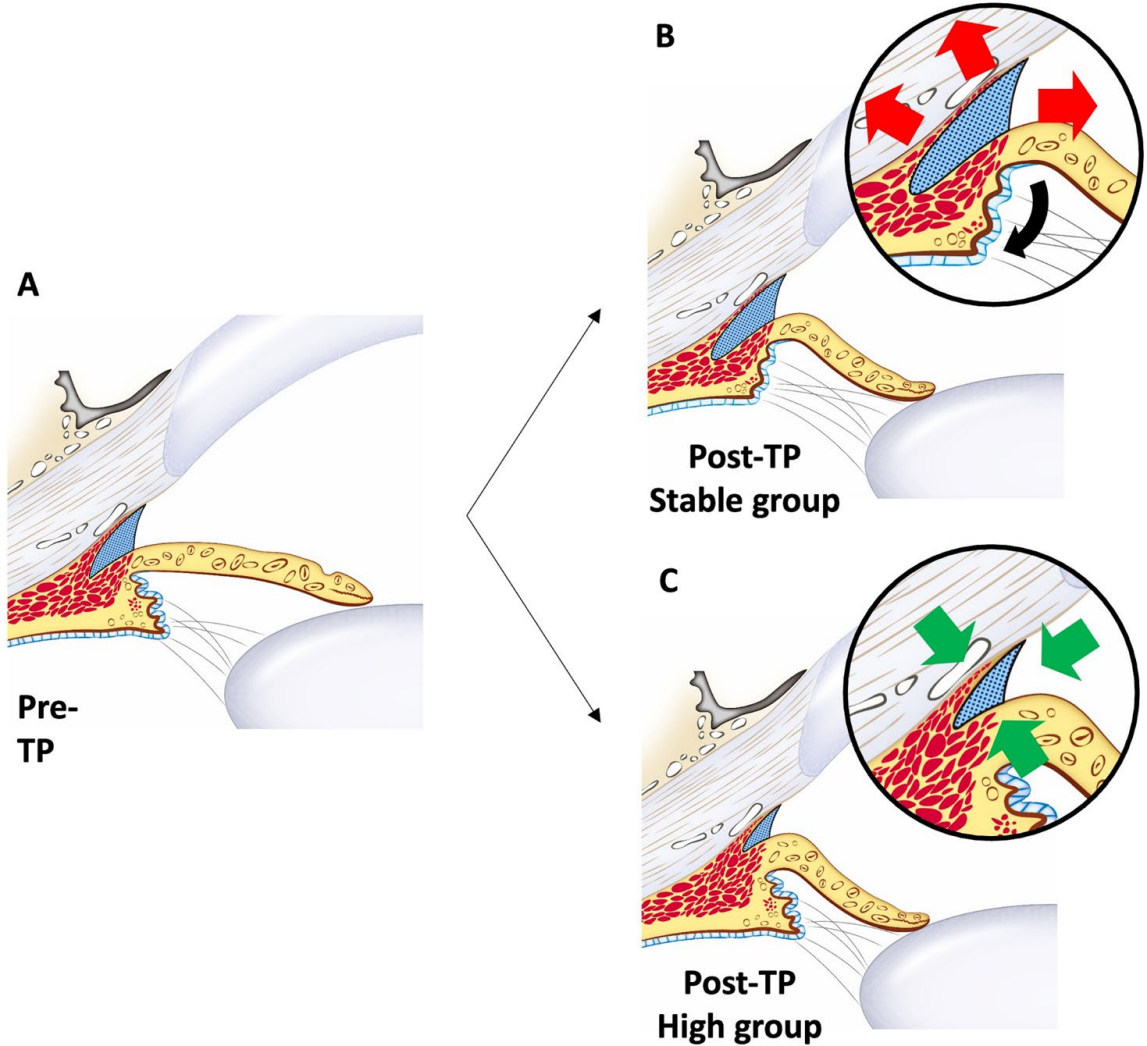
Ultrasound biomicroscopy (UBM) illustrations depicting anterior segment changes in canine eyes before and after topical tropicamide‐phenylephrine administration. (A) Pre‐TP: UBM image illustrating baseline anatomical structures before the application of 0.5% tropicamide‐0.5% phenylephrine. (B) Post‐TP, Stable Group: Following administration, pupil dilation compresses the ciliary cleft, but the ciliary body relaxes and moves posteriorly and outward, resulting in a relative enlargement of the ciliary cleft. (C) Post‐TP, High Group: In contrast, pupil dilation compresses the ciliary cleft, with no significant ciliary body relaxation, leading to a reduction in ciliary cleft dimensions. These findings highlight the differential effects of ciliary body dynamics on anterior chamber changes and intraocular pressure responses between the stable and high groups.

In conclusion, this study reveals that the increase in IOP following TP administration is associated with changes in the anterior chamber. The findings suggest that anatomical variations, particularly in the ciliary cleft and ciliary body dynamics, play a crucial role in the IOP response to mydriatic agents. Specifically, dogs in the high group exhibited less relaxation of the ciliary body and a reduction in CCA, indicating a predisposition to elevated IOP and a potential risk for glaucoma (Figure 6). Overall, this study contributes to veterinary ophthalmology by proposing a novel approach for predicting potential glaucoma risk, which could possibly facilitate earlier detection and more effective prevention strategies in canine patients.

## Author Contributions

**Donghee Kim:** conceptualization, data curation, formal analysis, methodology, visualization, writing – original draft, writing – review and editing. **Sang‐Eun Park:** conceptualization, data curation, formal analysis, investigation, methodology, writing – review and editing. **Haerin Yoon:** formal analysis, methodology. **Jiyi Hwang:** validation, visualization. **Ji Seung Jung:** validation, visualization. **Soohyun Kim:** writing – review and editing. **Kyung‐Mee Park:** funding acquisition, project administration, validation, writing – original draft, writing – review and editing.

## Ethics Statement

This study received ethical approval from the Institutional Animal Care and Use Committee (CBNUA‐1700‐22‐02). All animal owners or their representatives provided written informed consent for their pets' enrollment in the study, including the procedures and therapies undertaken, as well as for the publication of data and images derived from the study.

## Conflicts of Interest

The authors declare no conflicts of interest.

## Data Availability

The original contributions presented in the study are included in the article/Supporting Information; further inquiries can be directed to the corresponding author.
